# Multiple effects of electroporation on the adhesive behaviour of breast cancer cells and fibroblasts

**DOI:** 10.1186/1475-2867-12-9

**Published:** 2012-03-22

**Authors:** Viktoria N Pehlivanova, Iana H Tsoneva, Rumiana D Tzoneva

**Affiliations:** 1 Institute of Biophysics and Biomedical Engineering, Bulgarian Academy of Sciences, Acad. G. Bonchev Str., Bl. 21, Sofia 1113, Bulgaria

**Keywords:** Breast cancer cells, Fibroblasts, Actin cytoskeleton, Electroporation

## Abstract

**Background:**

Recently electroporation using biphasic pulses was successfully applied in clinical developments for treating tumours in humans and animals. We evaluated the effects of electrical treatment on cell adhesion behaviour of breast cancer cells and fibroblasts. By applying bipolar electrical pulses we studied short- and long-lived effects on cell adhesion and survival, actin cytoskeleton and cell adhesion contacts in adherent cancer cells and fibroblasts.

**Methods:**

Two cancer cell lines (MDA-MB-231 and MCF-7) and one fibroblast cell line 3T3 were used. Cells were exposed to high field intensity (200 - 1000 V/cm). Cell adhesion and survival after electrical exposure were studied by crystal violet assay and MTS assay. Cytoskeleton rearrangement and cell adhesion contacts were visualized by actin staining and fluorescent microscope.

**Results:**

The degree of electropermeabilization of the adherent cells elevated steadily with the increasing of the field intensity. Adhesion behaviour of fibroblasts and MCF-7 was not significantly affected by electrotreatment. Interestingly, treating the loosely adhesive cancer cell line MDA-MB-231 with 200 V/cm and 500 V/cm resulted in increased cell adhesion. Cell replication of both studied cancer cell lines was disturbed after electropermeabilization. Electroporation influenced the actin cytoskeleton in cancer cells and fibroblasts in different ways. Since it disturbed temporarily the actin cytoskeleton in 3T3 cells, in cancer cells treated with lower and middle field intensity actin cytoskeleton was well presented in stress fibers, filopodia and lamellipodia. The electrotreatment for cancer cells provoked preferentially cell-cell adhesion contacts for MCF-7 and cell-ECM contacts for MDA-MB- 231.

**Conclusions:**

Cell adhesion and survival as well as the type of cell adhesion (cell-ECM or cell-cell adhesion) induced by the electroporation process is cell specific. The application of suitable electric pulses can provoke changes in the cytoskeleton organization and cell adhesiveness, which could contribute to the restriction of tumour invasion and thus leads to the amplification of anti-tumour effect of electroporation-based tumour therapy.

## Background

Electroporation is a biophysical method, performed by the application of high voltage electrical pulses to cells *in vitro *or tissues *in vivo*, used to increase the cell's uptake of different molecules by permeabilization of the plasma membrane [1-4]. Most of the electropermeabilization protocols use unipolar electrical pulses [5-7], but recently the higher efficiency of biphasic pulses was confirmed [8-10] so they were successfully used in clinical developments for treating tumours in humans and animals [11-13] and for DNA transfection [8]. The field intensity and duration of the applied electrical pulses of the electroporation (electropermeabilization) can either reversibly open nanoscale pores on the cell membrane after which the cell can survive, or irreversibly open the cell membrane, after which the cell dies [14]. In cancer treatment, the reversible electroporation has been exploited to increase transport of chemotherapeutic drugs through the plasma membrane into the tumour cells. This process is called electrochemotherapy [1] and it is widely used for the treatment of accessible human tumours and tumour lesions [15-18]. Non-thermal ablation is a recently discovered new technique for treating inoperable tumours [19], which is based on irreversible electroporation of cells [20]. It is believed to affect only the cell membrane and no other structure in the tissue and in this way a direct electrical filed induced cancer cell death is achieved. Moreover, not as selective as electrochemotherapy, the thermal ablation can be used as a minimally invasive surgical procedure to ablate cancer tissue without the use of potentially harmful chemotherapeutic drugs.

Apart from the effect on cell membrane (to open nanoscaled pores), the applied external electric pulses demonstrate to be able to alter the cytoskeletal reorganization which affects the cell adhesion. For instance, changes in the cytoskeletal structure have been demonstrated during processes of electrofusion [21] and electrotransfer [22]. Actin cytoskeletal redistribution has been reported in directional cell electromigration induced by dc electrical field [23,24] and in electroporation-based therapies [25,26]. For example Kanthou et al. [25] studied the vascular effect of electropermeabilization as well as the changes in the cytoskeleton organization of primary endothelial cells and in the monolayer permeability. The results of Xiao et al., [26] which showed that the disruption of actin skeleton of cancer cells by application of electrical pulses, prevents cells from apoptosis and necrosis were very interesting too.

Using plated/adherent cells in the experimental model we can study the cells in their intact internal structure (cytoskeleton) and the results obtained in these cells are better comparable to real *in vivo *situations than the results from cell suspensions [27]. Comparing all data concerning actin cytoskeleton changes (how strong they can be and if they are reversible) in adherent cells induced by applied electrical pulses, it becomes visible that they depend mainly on the intensity of the applied field, electropulsation medium and cell type. For instance, it was shown that when culture medium was used during electroporation, the cytoskeletal structures [25] were best preserved. Yizraeli and Weihs [28] showed that fibroblasts were affected very weakly by the applied electrical field in comparison to MDA-MB- 231 cells.

In adherent cells, the basic actin-rich cell-extracellular matrix (ECM) ensembles are stress fibers, lamellipodia and filopodia, which play an important role in cell attachment and migration [29-31]. Actin can also be arranged into peculiar dot-like structures called podosomes which perform a role in cell migration and motility and in ECM degradation [32,33]. In cancer cells the presence of adhesion contacts is not a prerequisite for growth and survival [34]. Among the main features of the cancer cells are the breakdown of adherent connections (cell-cell contacts) and also the cytoskeleton organization (cell-ECM contacts) [35]. The change in the adhesive behaviour of cancer cells determines their modified morphology and migration behaviour and predetermines their invasive properties during all stages of tumourogenesis [36,37]. Thus, changing the cell's adhesion ability by electroporation it could be a very important prerequisite to inhibit cancer cells motility, invasion and metastasis.

The surrounding stroma of many tumours, for instance breast tumours, consists mainly of fibroblasts [38], which play prominent role in the development and progression of the tumour [39-41]. Therefore stromal fibroblasts have to be considered as a possible target in electropulse tumour treatment and may affect the outcome of any treatment applied to a definite region [28].

Regardless of some articles, concerning the changes in the cell cytoskeleton provoked by applied electrical pulses [21,22,25], little is known about the influence of these changes during the process of electroporation on actin cytoskeleton of adherent cancer cells.

Therefore, the aim of the present paper is to study the effect of the applied biphasic electrical pulses (200 - 1000 V/cm) on the adhesive behaviour of two breast cancer cell lines and a non-transformed fibroblast cell line in order to elucidate the effect of electrical field on tumour progression. MDA-MB-231 was chosen as a cell model for invasive and metastatic breast cancer cells and MCF-7 cell line was used as an example of a fast growing non-invasive breast cancer cell line. The non-tumorogenic cell line 3T3 (mouse fibroblasts) was chosen to present the somatic fibroblasts surrounding the tumour.

## Materials and methods

### Chemicals and proteins

Propidium iodide (PI) (Sigma-Aldrich Company Ltd, St. Louis, Missouri, USA) was prepared in phosphate buffered saline (PBS), pH 7.4 in a concentration of 0.1 mM.

Crystal violet was from Sigma, (St. Louis, Missouri, USA) and was used in concentration of 0.1% in PBS, pH 7.4. BODIPY 558/568-conjugated phalloidin (B3475, Invitrogen GmbH, Karlsruhe, Germany) was used in a concentration of 0.132 μM. Human plasma Fibronectin (FN, Roche Applied Science, Penzberg, Germany) in a concentration of 20 μg/ml was used for coating of surfaces for the investigation with cells.

### Cell lines

MDA-MB-231 (ATCC, Manassas, VA, USA) were grown in RPMI 1640 medium (PAA, The Cell Culture Company, Cat. № E15-039, Germany) with 10% fetal calf serum (FCS) and supplements (insulin, L-glutamine, sodium pyruvate, antibiotic, NEAA (non essential amino acids) at 37°C, 5% CO_2 _and humidified atmosphere.

MCF-7 (ATCC, Manassas, VA, USA) cell line was cultivated in modified Eagle's medium (DMEM), (PAA: The Cell Culture Company, Cat. № E15-009, Germany) containing 10% FCS and supplements L-glutamine, sodium pyruvate, antibiotic, NEAA at 37°C, 5% CO_2 _and humidified atmosphere. 3T3 cell line (mouse fibroblasts) (ATCC, Manassas, VA, USA) was cultivated in Eagle's Minimum Essential Medium (MEM), (PAA, The Cell Culture Company, Cat. № E15-825, Germany) containing 10% FCS and supplements (L-glutamine, sodium pyruvate, antibiotic, NEAA at 37°C and 5% CO_2_.

### Cell electroporation

**Figure 1:**
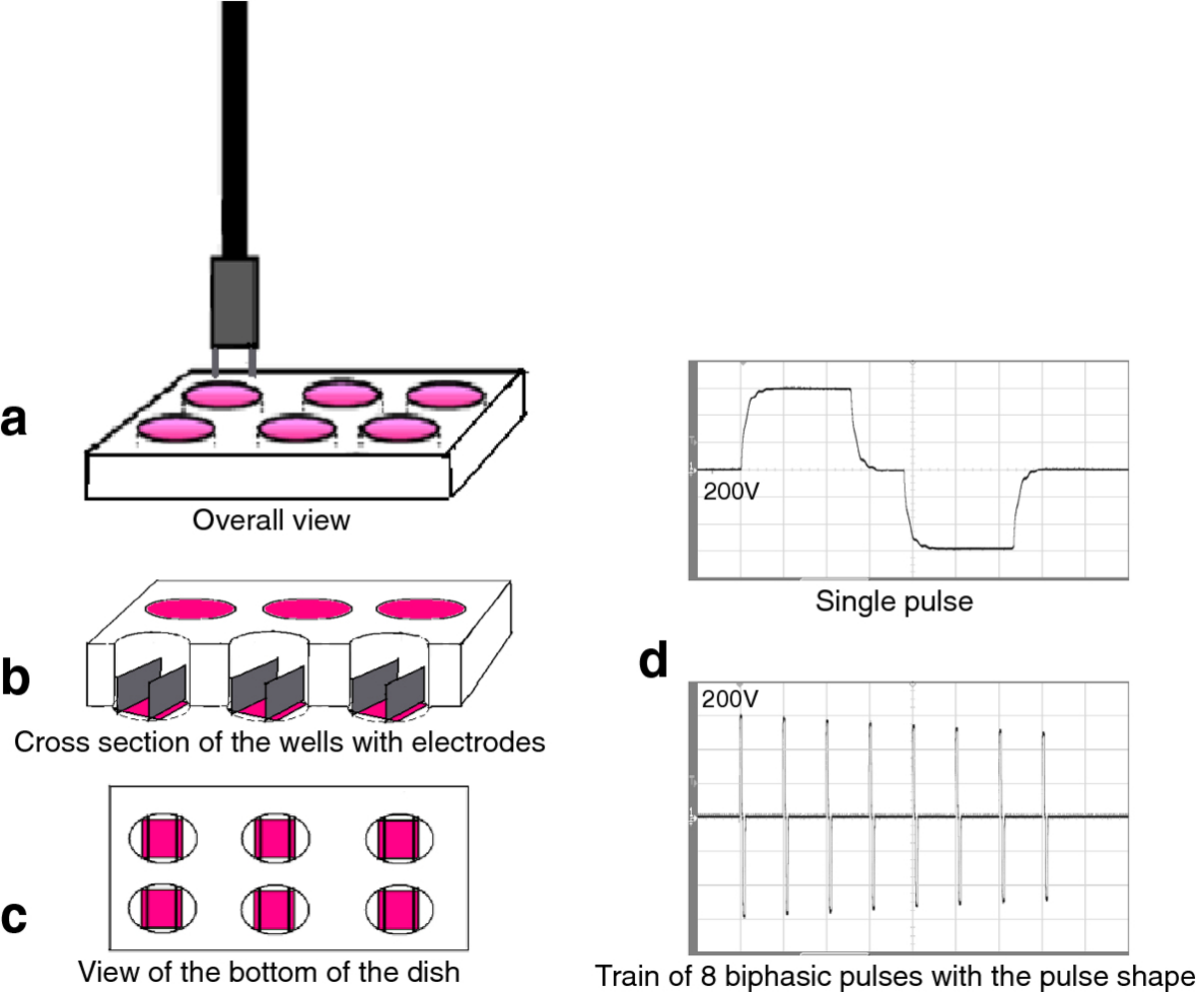
**Scheme of the experimental setup and the shape of the electric pulses**. **a**), **b**) and **c**) - experimental setup, **d**) voltage recording with biphasic pulses.

Chemipulse III electroporation apparatus was produced at the Institute of Biophysics and Biomedical Engineering, Bulgarian Academy of Sciences, Sofia, Bulgaria. The following electrical parameters were used for the experiments - 8 biphasic pulses; 50 + 50 μs with 20 μs interval between both phases and pause between bipolar pulses of 880 μs, the voltage was between 200 - 1000 V. At that field conditions Daskalov and Bankov have proved that the effectiveness of the electroporation is high [42]. The apparatus was supplied with a battery and during the manipulations it was not connected to the mains supply. The induction of 200 - 1000 V voltage was done in two parallel stainless steel electrodes (Figure 1) with a 10 mm distance between them and a length of 22 mm (when cells are cultivated on cover glasses) or 9 mm (when cells are cultivated directly on the bottom of 24 well plates). A uniform electric field was generated by approximation ignoring edge effects. A scheme of the experimental dish and shape of the electrical pulses is shown in Figure 1.

When the cells are 80 - 90% confluent they were harvested with 0.05% trypsin/0.6 mM ethylenediaminetetraacetic acid (EDTA) (Sigma, Deisenhofen, Germany) at 37°C. Trypsin was neutralized with FCS. The cells were centrifugated (at 1 × 10^3 ^RPM for 5 min) and resuspended in the appropriate cultivation medium. They were seeded directly on the bottom of 24 well plates (Greiner Bio-One GmbH, Solingen, Germany) or on cover slides (18/18 mm, Superior-Marienfeld, Germany), which were coated with FN. Before protein coating the cover slides were rinsed once with 70% ethanol and twice in sterile distilled H_2_O and placed in 6 well plates (Greiner Bio-One GmbH, Solingen, Germany). The cells were incubated for 24 hours at 37°C and 5% CO_2 _to reach stable adhesion prior to the electrical treatment. Electroporation was carried out in basal cell medium (without phenol red and supplements). Immediately after electroporation the basal cell medium was replaced by supplemented medium with 10% FCS for the additional incubations.

### Determination of electro-permeabilization

The electro-permeabilization of plasma membrane was measured by cellular uptake of PI. 3T3 cells at cell density of 1.5 × 10^5 ^cells ml^-1 ^were cultivated on cover glasses in 6 well plates. After 24 hours the adhered cells were electroporated in a basal cell medium containing 0.1 mM PI (Sigma, Deisenhofen, Germany) under the above electrical parameters. After the exposure to electrical pulses, the cells were incubated for 15 min at 37°C. This incubation time was shown to be the optimal, since it allows resealing of the plasma membrane and it does not affect cell viability due to the evaporation of the medium and lack of nutrients [43]. To visualize the PI uptake, the cover glasses were washed with PBS, pH 7.4 and then were fixed with 3% paraformaldehyde (PFA) for 15 minutes at room temperature. After three washes with PBS and distilled H_2_O, the slides were mounted on objective glasses using Mowiol and were visualized using fluorescent inverted microscope (Leica DMI3000 B, Leica Microsystems GmbH, Germany) with objective HI PLAN 40×/0.50, filter set I3 S and light source from Hg 100 W lamp. The images were taken by camera Moticam 2500, 5.0 M Pixels USB 2.0. For each used voltage 5 images from different fields of the sample were produced. The middle section of the cover glass, which is situated in the centre of the electrical field, was examined.

### Cell adhesion (crystal violet assay)

Cell adhesion assay was adapted to that described by Hernandez et al. [44]. 200 μl cell suspension with density of 1.5 × 10^5 ^cells ml^-1 ^and 10% FCS were seeded in each of the 24 well plates (Greiner Bio-One GmbH, Solingen, Germany). After 24-hour incubation at 37°C and 5% CO_2 _the adhered cells were electroporated in a basal cell medium. No electrical pulses were applied to the cells in the control. After the electrical treatment, the cells were incubated for additional 2 and 24 hours. The well plates were washed three times with PBS, pH 7.4 to remove the non-adhered cells and then the adhered cells were fixed with 500 μl 3% solution of PFA for 20 minutes at room temperature. The cells were washed twice with distilled H2O, stained with 0.1% solution of crystal violet for 20 minutes at room temperature, washed again with distilled H_2_O and dried for 24 hours at 37°C. 100 μl 0.1 M HCl was added in each well. The relative number of the adhered cells was defined colorimetrically by the intensity of the solution of crystal violet at 630 nm using a microplate reader (Multiskan Spectrum, Thermo Electron Corp., Finland). Three independent experiments with three repeats were performed for each cell line.

### Cell survival

The survival of cells after electrotreatment was determined as described by Cemazar et al. [45]. We used CellTiter 96 AQ_ueous _One Solution Cell Proliferation MTS assay (Promega, Madison, WI, USA) to determine cell survival. Cells were electrotreated as described for cell adhesion assay. After the electrical treatment, the cells were incubated for 2 and 24 hours. Then, 50 μl of MTS reagent was added directly to the adherent cells. They were incubated for 2 hours at 37°C and recorded the absorbance at 490 nm with 96-well plate reader Tecan Infinite F200 PRO (Tecan Austria GmbH, Salzburg). The survival of the cells treated with different electrical field intensities was presented as a relative number of adhered cells (O.D. at 490 nm). Three independent experiments were performed for each cell line.

For both cell adhesion and survival experiments a fraction of cells was calculated, where the cells were under the influence of the electrical field in the sample. We assumed that the cells were randomly adhered on the bottom of the well. The electrodes used for electroporation (the distance between the electrodes was 10 mm and the length of the electrodes used for these experiments was 9 mm) cover a frame between them with area (S_e_) of 90 mm^2^. The total surface area (S_t_) of the bottom of the 24 well plates was equal to 132.66 mm^2^. Thus, approximately 70% of all cells adhered on the well were under the applied electrical field.

### Actin staining

3T3, MDA-MB-231 and MCF-7 with cell density of 1.5 × 10^5 ^cells ml^-1 ^were cultivated on cover glasses (18/18 mm) placed in 6 well plates. After 24-hour incubation the cells were electroporated in a basal cell medium and were cultivated additionally for a period of 2, 24 and 48 hours in full cell medium. After the incubation period, non-adhered cells were removed by triple rinsing with PBS, pH 7.4. The adhered cells were fixed with 1 ml 3% solution of PFA for 15 minutes at room temperature. The fixed cells were permeabilized using 1 ml 0.5% solution of Triton X-100 for 5 minutes and then incubated with 1 ml 1% solution of bovine serum albumin (BSA) for 15 minutes. The samples were washed three times with PBS, pH 7.4 and then incubated for 30 minutes at room temperature with BODIPY 558/568 phalloidin. Again, the samples were washed three times with PBS and once with distilled water, and then were installed on objective glasses by Mowiol. Preparations were analyzed using inverted fluorescent microscope (Leica DMI3000 B, Leica Microsystems GmbH, Germany) with object HCX PL FLUOTAR 63×/1.25 oil.

## Results and Discussion

### Degree of cell electroporation

**Figure 2:**
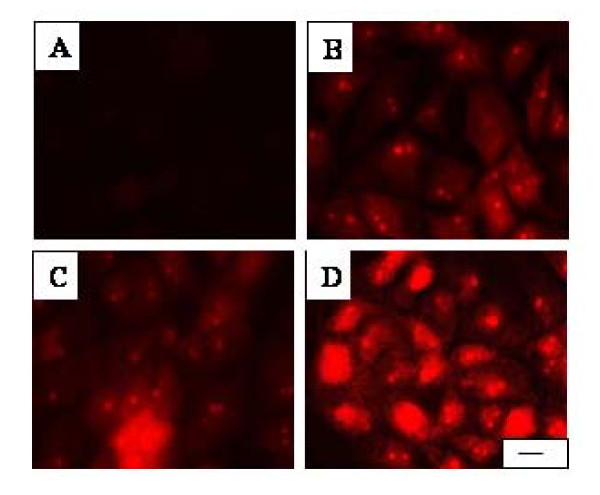
**3T3 cells electroporated in the presence of propidium iodide and incubated for 15 minutes**. (**A**) control sample (non-porated cells); (**B**) cells electroporated at 200 V/cm; (**C**) at 500 V/cm; (**D**) at 1000 V/cm. The bar is 40 μm. The pictures were representative from five others made in different fields in central part of the sample.

Penetration of PI into the cells was used to define the success of permeabilization of the cell membrane by the process of electroporation. The results showed membrane permeabilization at all three intensities of the electrical field (Figure 2, B-D). For PI staining pictures were made at the same parameters of the fluorescent microscope for the exposure of the sample as time of exposure, brightness, contrast, etc. It was visible that cells treated with 200 V/cm and 500 V/cm were less electroporated than those at 1000 V/cm (Figure 2). The results showed that the content of PI in the cells increase at a higher electrical filed intensity. Each picture in Figure 2 is a representative of five different pictures taken at different electrical intensity.

### Cell adhesion and survival

Cell adhesion is an important process in cancer insemination. We have tested short- and long-lived effects on cell adhesion caused by electropermeabilization.

**Figure 3:**
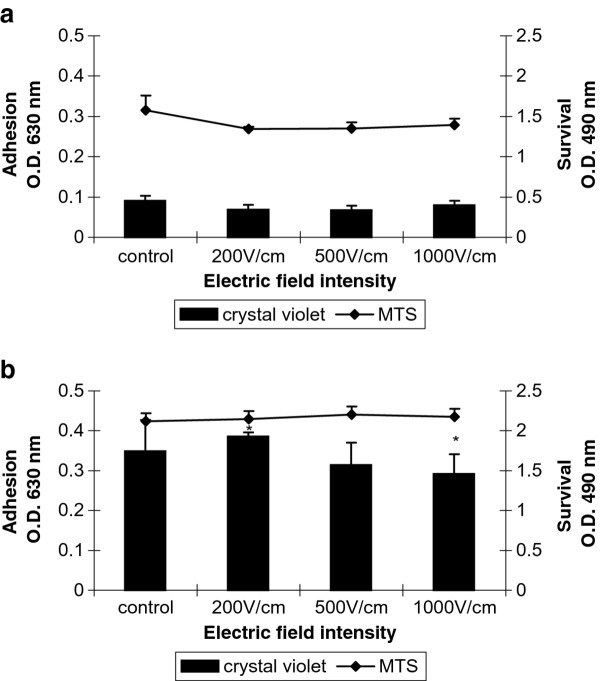
**Cell adhesion and survival of 3T3 cells**. Cells are incubated 24 h prior electroporation for good cell adhesion. 2 h (**A**) and 24 h (**B**) after electrical treatment are measured cell adhesion by crystal violet assay and cell survival by MTS assay. Data are means ± SD of seven replicates. The error bars are two standard deviations in total height. The statistics was performed by one-way analysis using the Tukey-Kramer post test (* *p <*0.05).

**Figure 4:**
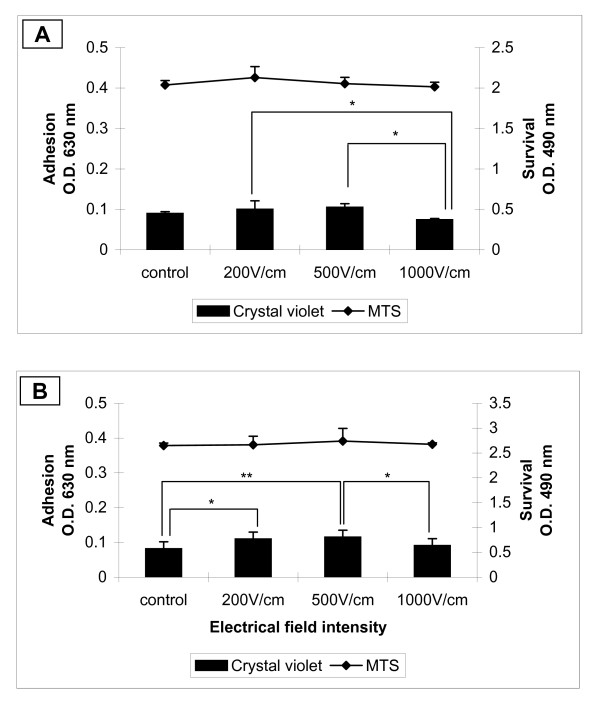
**Cell adhesion and survival of MDA-MB-231 cells**. Cells are incubated 24 h prior treatment for good cell adhesion. 2 h (**A**) and 24 h (**B**) after electrical treatment are measured cell adhesion by crystal violet assay and cell survival by MTS assay. Data are means ± SD of five replicates. The error bars are two standard deviations in total height. The statistics was performed by one-way analysis using the Tukey-Kramer post test (*p <0.05 and **p <0.01).

**Figure 5:**
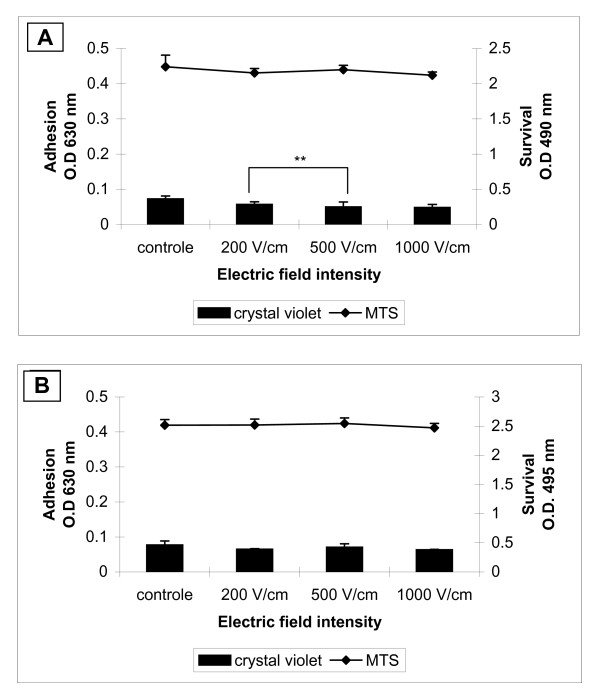
**Cell adhesion and survival of MCF-7 cells**. Cells are incubated 24 h prior treatment for good cell adhesion. 2 h (**A**) and 24 h (**B**) after electrical treatment are measured cell adhesion by crystal violet assay and cell survival by MTS assay. Data are means ± SD of five replicates. The error bars are two standard deviations in total height. The statistics was performed by one-way analysis using the Tukey-Kramer post test (**p <0.01).

The colorimetric method of crystal violet staining was used for quantitative estimation of cell adhesion and by MTS assay the cell survival after electropermeabilization was determined. All cells were cultivated 24 h prior electroporation on FN coated cover glasses. In Figure 3 adhesion and survival of electroporated 3T3 cells after 2 (Figure 3A) and 24 (Figure 3B) hours are shown. Cell adhesion of 3T3 cells was not affected significantly by the electrical treatment (Figure 3) over the entire examined time. Decrease in cell adhesion was detected only at 1000 V/cm 24 hours after electroporation in comparison to those at 200 V/cm (asterisks in Figure 3B). The replication of cells was not affected by electropermeabilization since after 24 hours the relative number (O.D. at 630 nm) of non-treated cells (control) and electrotreated cells increased between 30% - 40%. Minor decrease of cell survival of all treated cells 2 hours after electroporation was detected. There was no difference in the cell survival between treated cells and the control 24 hours later. The invasive tumour cell line MDA-MB-231 showed different cell behaviour when treated with high voltage electrical pulses (Figure 4A and 4B). It is well known that MDA-MB-231 cells exhibit week adhesive behaviour in general [46]. Two hours after the electrical treatment, cell adhesion at the higher field intensities (1000 V/cm) decreased significantly compared to the control and cells treated with 200 V/cm and 500 V/cm. Cell survival did not decrease significantly when increasing the intensity of the electrical pulses (Figure 4A). Surprisingly, even after 24-hour-electrotreatment cell adhesion at 200 V/cm and 500 V/cm showed some increase (comparing to the control) and only the cell adhesion at 1000 V/cm was diminished in comparison to those at 500 V/cm (Figure 4B). Cell survival after 24 hours seemed to be not affected by electroporation. The non-invasive tumour cell line MCF-7 showed general tendency to decreasing of cell adhesion 2 hours after electroporation (Figure 5A). The adhesion was getting lower with the intensity increase of the electrical field and reached the minimum at 1000 V/cm (Figure 5A). The trend of the cell survival resembles the one of the 3T3 cells: the cell survival slowly decreases. After 24 hours the cell adhesion and survival were not affected by electrotreatment (Figure 5B). Our results are in agreement with Yizraeli and Weihs [28], who showed that fibroblasts is not affected significantly by electrical treatment in comparison to cancerous cells (MDA-MB-231). In general, for two cancer cell lines the electropermeabilization seems to slower down the cell replication since 24 hours later there was no significant increase in the relative cell number. In contrast to the work of Cemazar et al., [45] which showed a significant decrease in MCF-7 cell survival between 200 V/cm and 1000 V/cm, our results did not indicate a decrease in cell adhesion and survival between the above values of the electrical field intensity. Most probably the preadsorption step with FN is the factor which plays a protective role for cell adhesion and survival against the electrotreatment.

### Actin cytoskeleton

It is well known that the actin cytoskeleton plays a fundamental role in cell adhesion, migration and growth and in cancerogenesis these processes are significantly unregulated [47]. The investigation of the influence of high electrical pulses on the organization of actin cytoskeleton was conducted with two breast tumour cell lines and one non-transformed cell line - 3T3 fibroblasts. The alteration in actin cytoskeleton was followed up to 48 hours after the electrotreatment in order to monitor how stable the changes are.

#### a) 3T3 cells

**Figure 6:**
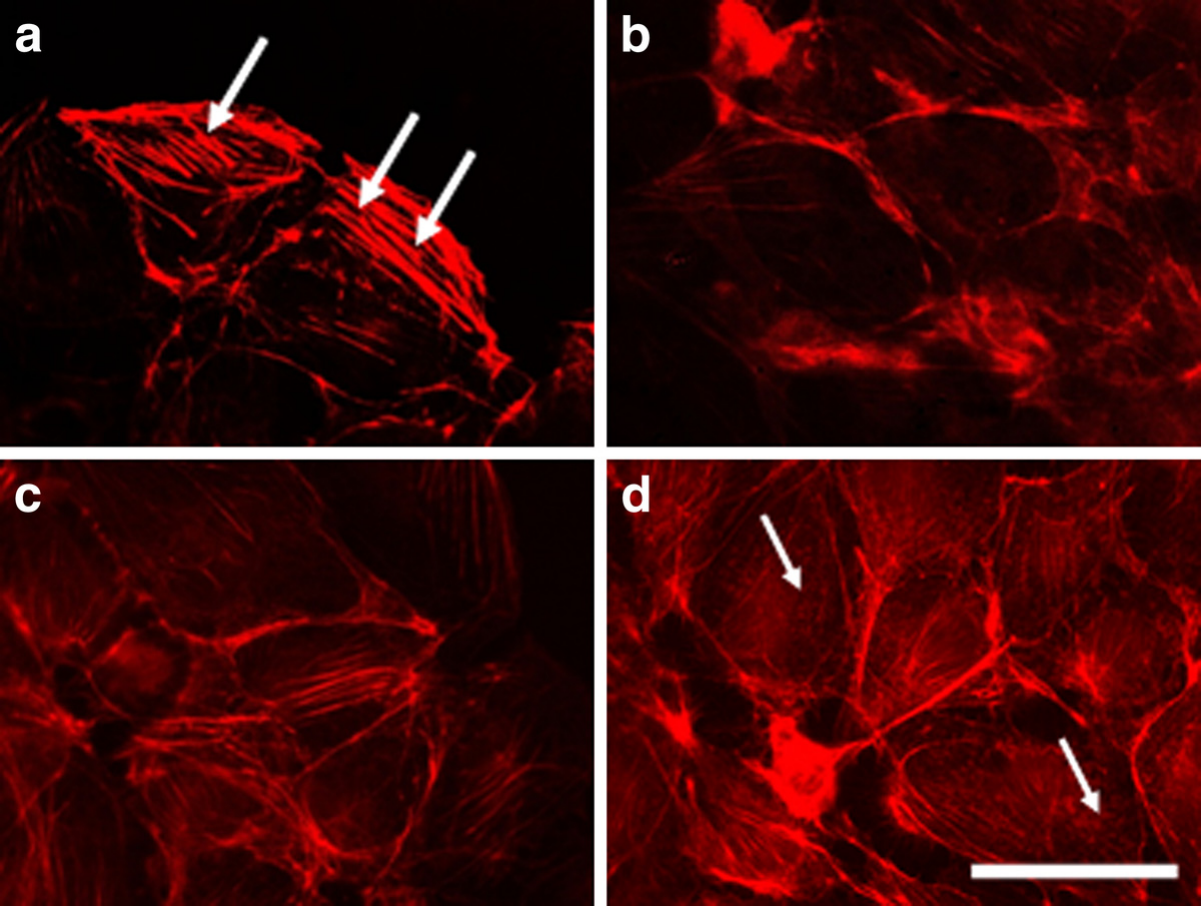
**3T3 cells incubated 2 h**.** after electroporation and stained for actin.** (**A**) control (non-electrotreated cells), (**B**) cells electroporated with 200 V/cm, (**C**) with 500 V/cm, (**D**) with 1000 V/cm. Bar is 50 μm.

Before the electroporation 3T3 cells showed typical fibroblast-like morphology with well pronounced intact actin filaments forming stress fibers along the whole cell body (arrows in Figure 6A). After being electroporated with different intensities the organization of cytoskeleton was changed (Figure 6B-D). The actin filaments decreased in number and became thinner as the effect was amplified with the increasing of the field intensity. At 1000 V/cm the cytoskeleton was visible only in the cell periphery (Figure 6D) and at the same time dot-like structures of actin forming a ring toward the cell periphery (podosomes) were observed (arrows in Figure 6D). Since podosomes are known mainly for their proteolytic function on ECM, we could consider that the application of high voltage increases the degradation function of cells. In general, the results indicate a reduced strength of cell adhesion.

**Figure 7:**
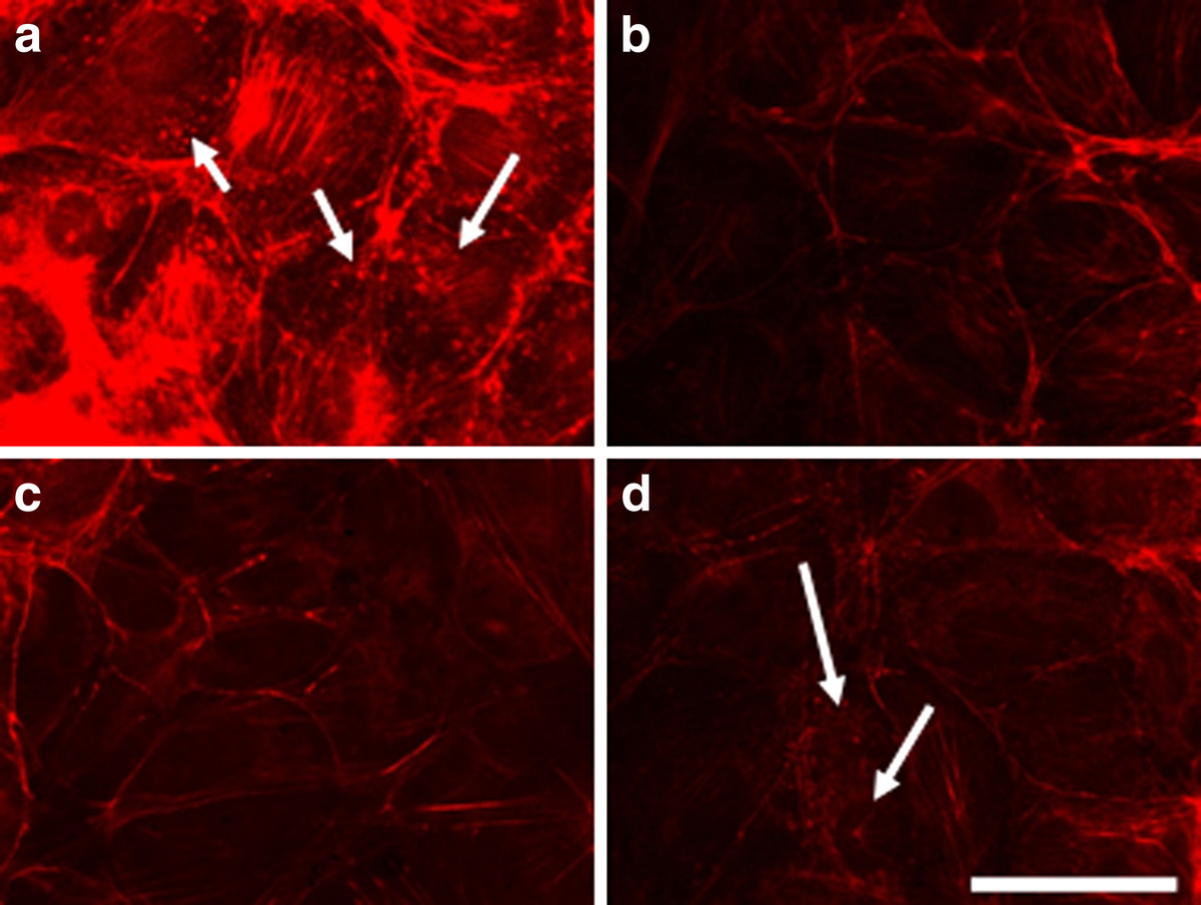
**3T3 cells incubated 24 h**. **after electroporation and stained for actin.** (**A**) control, (**B**) cells electroporated with 200 V/cm, (**C**) with 500 V/cm, (**D**) with 1000 V/cm. Bar is 50 μm.

**Figure 8:**
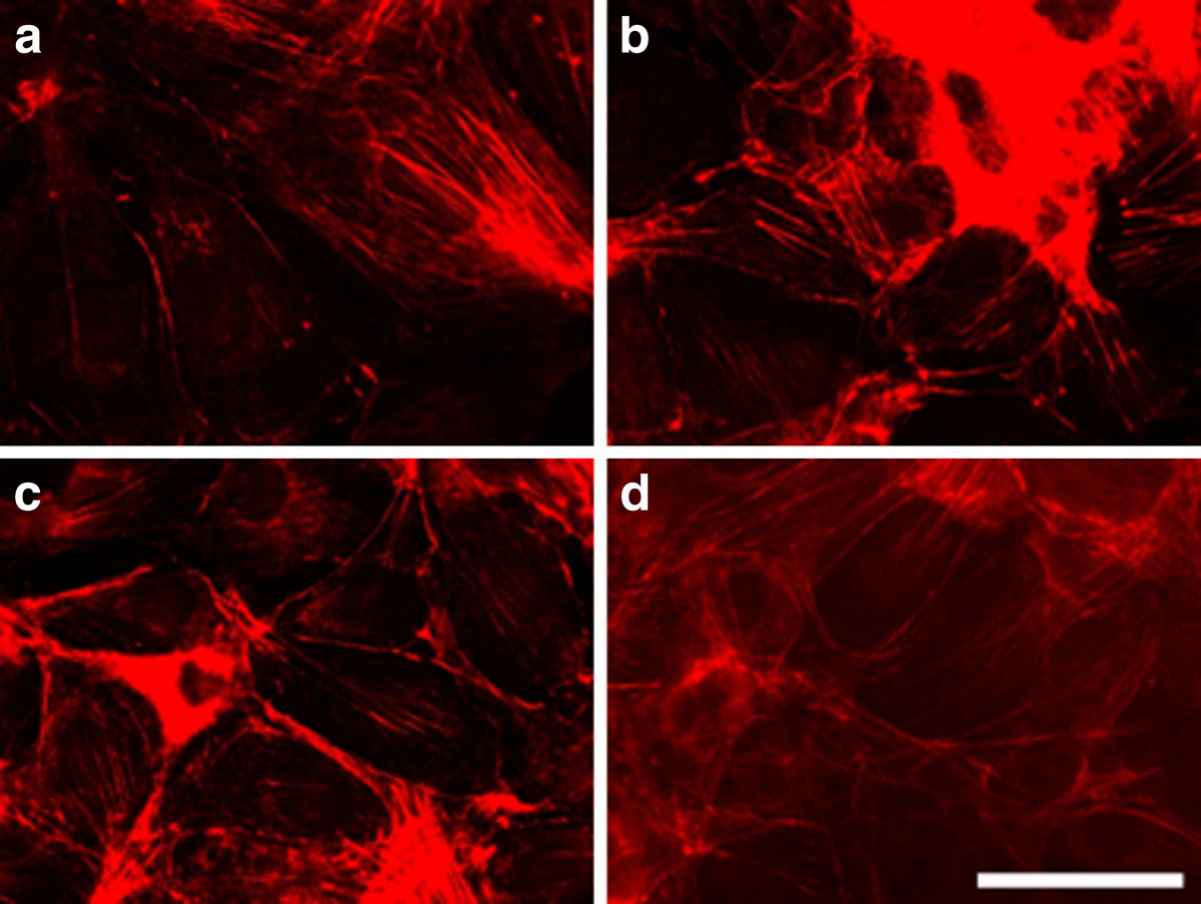
**3T3 cells incubated 48 h after electroporation and stained for actin**. (**A**) control, (**B**) electrotreated cell with 200 V/cm, (**C**) with 500 V/cm, (**D**) with 1000 V/cm. Bar is 50 μm.

After 24 hours the effect of electroporation faded. The actin filaments were reconstructed faster in cells treated with the lowest voltage - 200 V/cm (Figure 7B). Podosomes were still visible in the cells treated with 1000 V/cm (arrow in Figure 7C), but surprisingly they appeared in non-electrotreated cells (arrows in Figure 7A). Probably the podosome formation was initiated with exhausting the serum in the cell medium with time [48]. The recovery process of the intact actin cytoskeleton (the formation of stress fibers) 48 hours after electrotreatment seemed completed for the cells treated with 200 V/cm and 500 V/cm (Figure 8B and 8C), while the actin stress fibers in 3T3 cells treated with 1000 V/cm remained pale and fine (Figure 8D). From the presented results it could be concluded that the exposure of 3T3 cells to high voltage pulses leads to a fast but temporary disturbance of the actin filaments and the process is field intensity-dependent. Our findings are in agreement with other conducted experiments concerning the influence of the electroporation (mainly at the lower intensity range of 200 V/cm) on the disturbance of the cytoskeleton structures of nontransformed cells as fibroblasts [49] and endothelial cells [25]. In contrast to the complete recovery of cytoskeleton of the cells treated with 200 V/cm and 500 V/cm we show here that by using the typical intensities for electrochemotherapy - 1000 V/cm, the recovery of cell adherent contacts remains uncompleted. We observe a new fact - an increased proteolytic degradation potential of the electrotreated cells. This fact needs to be further proved if it is mainly caused by application of the electrical field.

The disturbance of the cytoskeleton of the adherent cells leads to changes in cell attachment, diminished cell motility and survival. The dominant type of stromal cells surrounding each tumour is from fibroblast origin [38]. Many investigations conducted *in vitro *and/or *in vivo *show that fibroblasts can support the growth of tumour cells [39-41]. We could suggest that the destabilization of actin cytoskeleton of the fibroblasts under the influence of high electrical pulses could lead to an additional positive effect of the applied electrochemotherapy leading to restriction of tumour expansion.

#### b) MDA-MB-231

**Figure 9:**
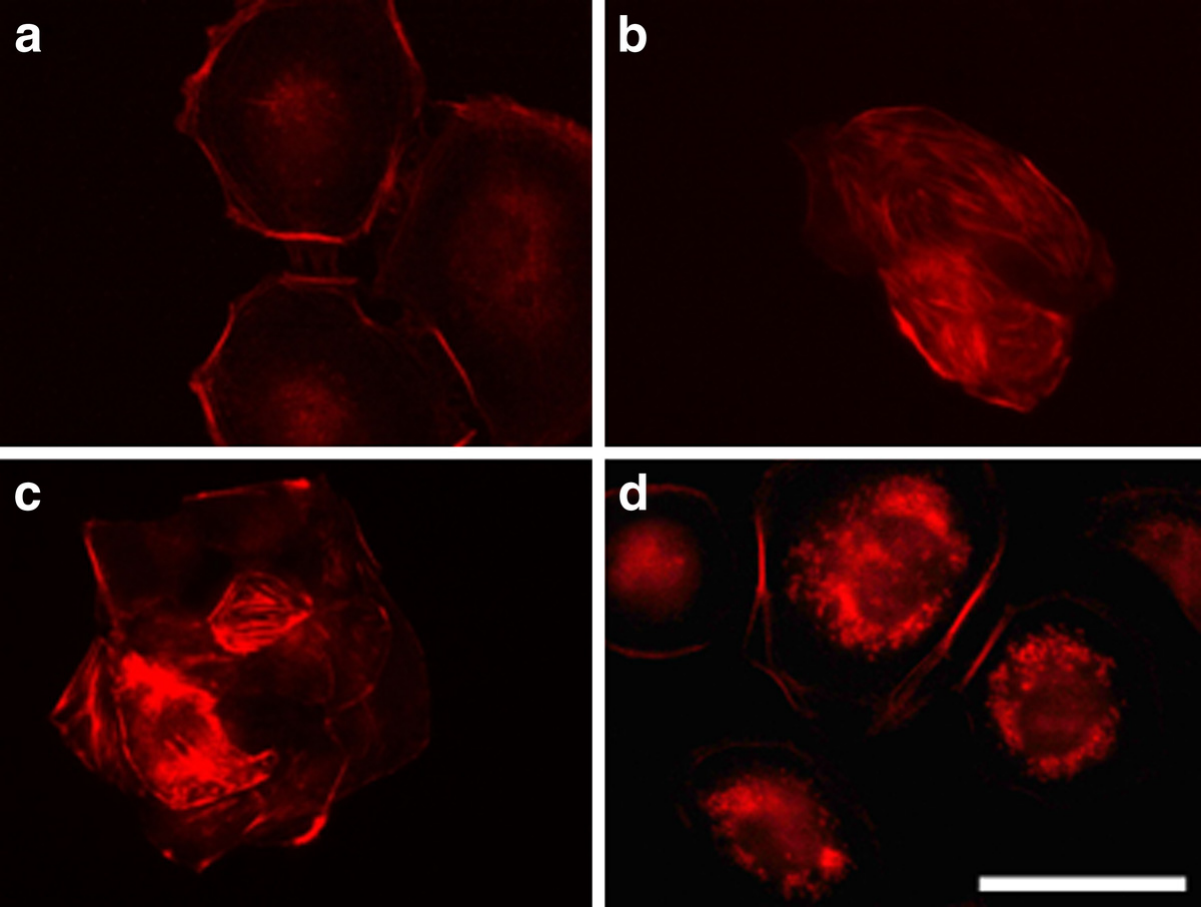
**MDA-MB-231 cells incubated 2 h after electroporation and stained for actin**. (**A**) control, (**B**) electrotreated cells with 200 V/cm, (**C) **with 500 V/cm, (**D**) with 1000 V/cm. Bar is 50 μm.

**Figure 10:**
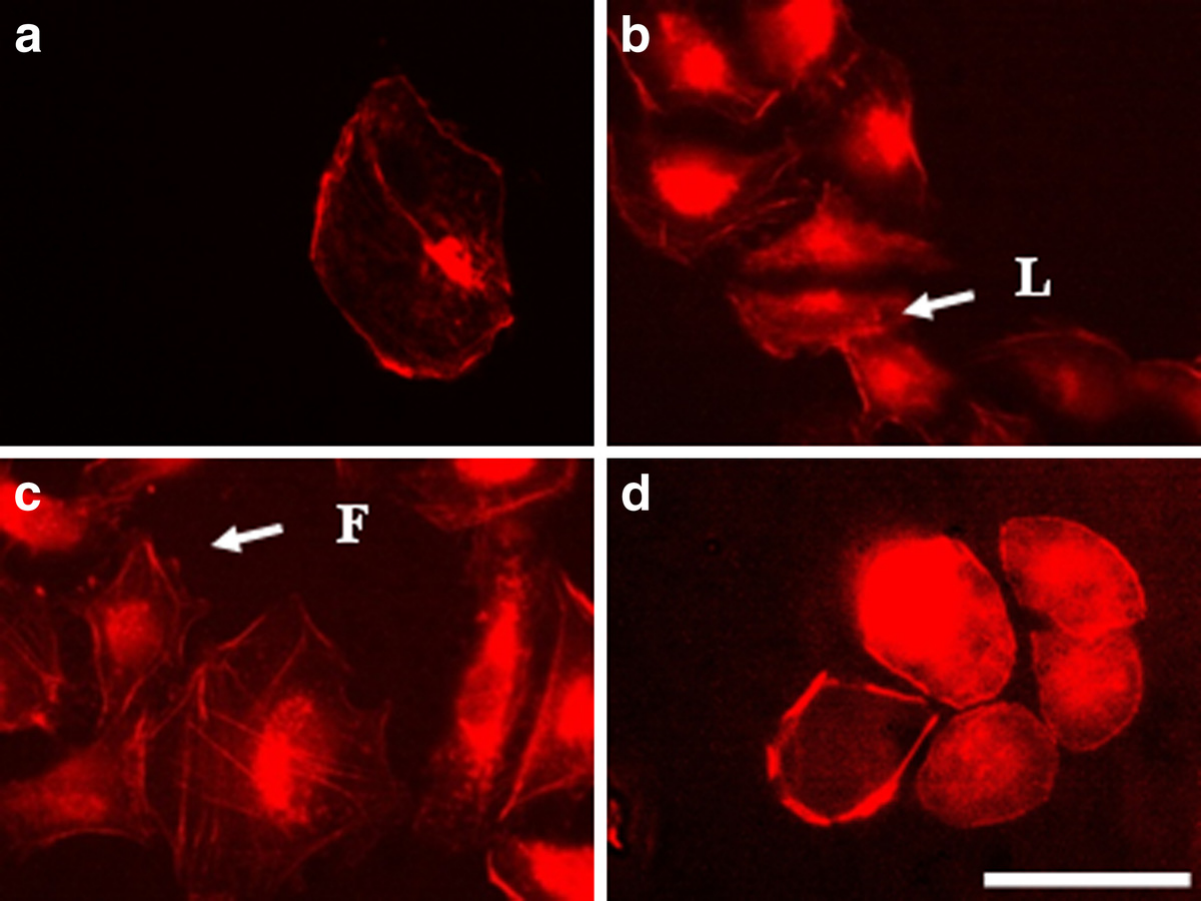
**MDA-MB-231 cells incubated 24 h after electroporation and stained for actin**. (**A**) control, (**B**) electrotreated cells with 200 V/cm, (**C**) with 500 V/cm, (**D**) with 1000 V/cm. Bar is 50 μm.

**Figure 11:**
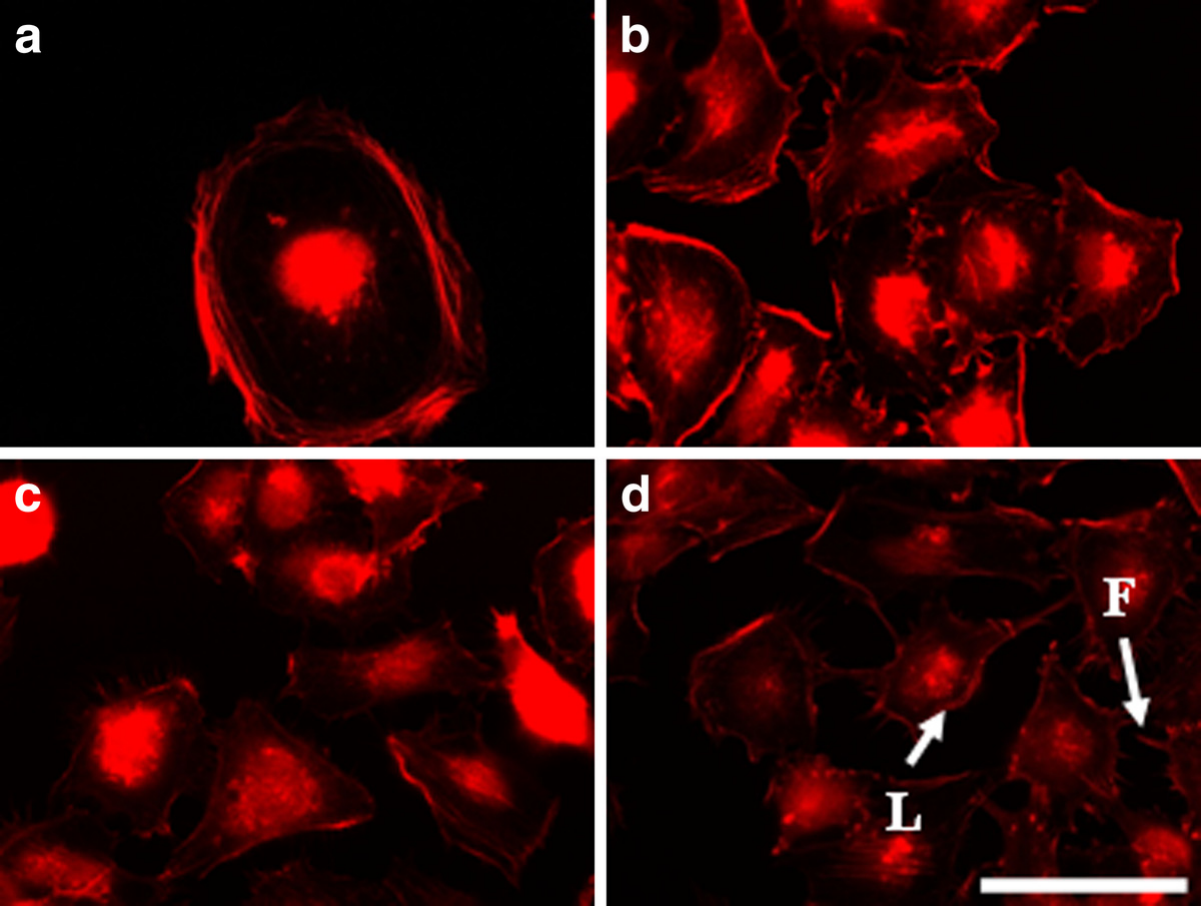
**MDA-MB-231 cells incubated 48 h after electroporation and stained for actin**. (**A**) control, (**B**) electrotreated cells with 200 V/cm, (**C**) with 500 V/cm, (**D**) with 1000 V/cm. L - lamellipodia, F - Filopodia. Bar is 50 μm.

The electroporation with 200 V/cm and 500 V/cm of highly invasive breast cell line MDA-MB- 231 after 2 hours led to formation of well visible actin stress fibers along the cells, which were organized in aggregates (Figure 9B and 9C). The formation of aggregates could be a result of the increased cell-cell contacts caused by increased Ca^2+^influx after the electrical treatment [50,51]. At the same time the electrical pulses with intensity of 1000 V/cm did not cause formation of aggregates (Figure 9D). Actin cytoskeleton in the control and in 1000 V/cm treated cells is organized peripherally (Figure 9A and 9B). Cells treated with 200 V/cm and 500 V/cm (Figure 10B and 10C) showed stabilized actin filaments (stress fibers along the cell body) and lack of cell aggregates 24 hours later. In addition we found actin structures as lamellipodia (arrows in Figure 10B) and filopodia (arrows in Figure 10C) which are both a sign for adhesive and migratory potential of the cells. The cells treated with 1000 V/cm showed more rounded shape with non-well-pronounced actin stress fibers (Figure 10D). The presence of these two cytoskeleton structures (filopodia and lamellipodia) was even stronger pronounced for cells treated with 200 V/cm and 500 V/cm (Figure 11B and 11C) 48 hours later, and also they appeared in the cells treated with 1000 V/cm (Figure 11D). It is important to note that new investigations show the coexistence of both lamellipodial and filopodial protrusions is an evidence of poorly migrating or non-motile cells, which usually causes cell migration arrest [52]. Overall, the results for MDA-MB-231 cells showed a tendency for an increase of cell adhesion (cell-substrate adhesion) with time mainly for cells treated with 200 V/cm and 500 V/cm.

#### c) MCF-7 cells

**Figure 12:**
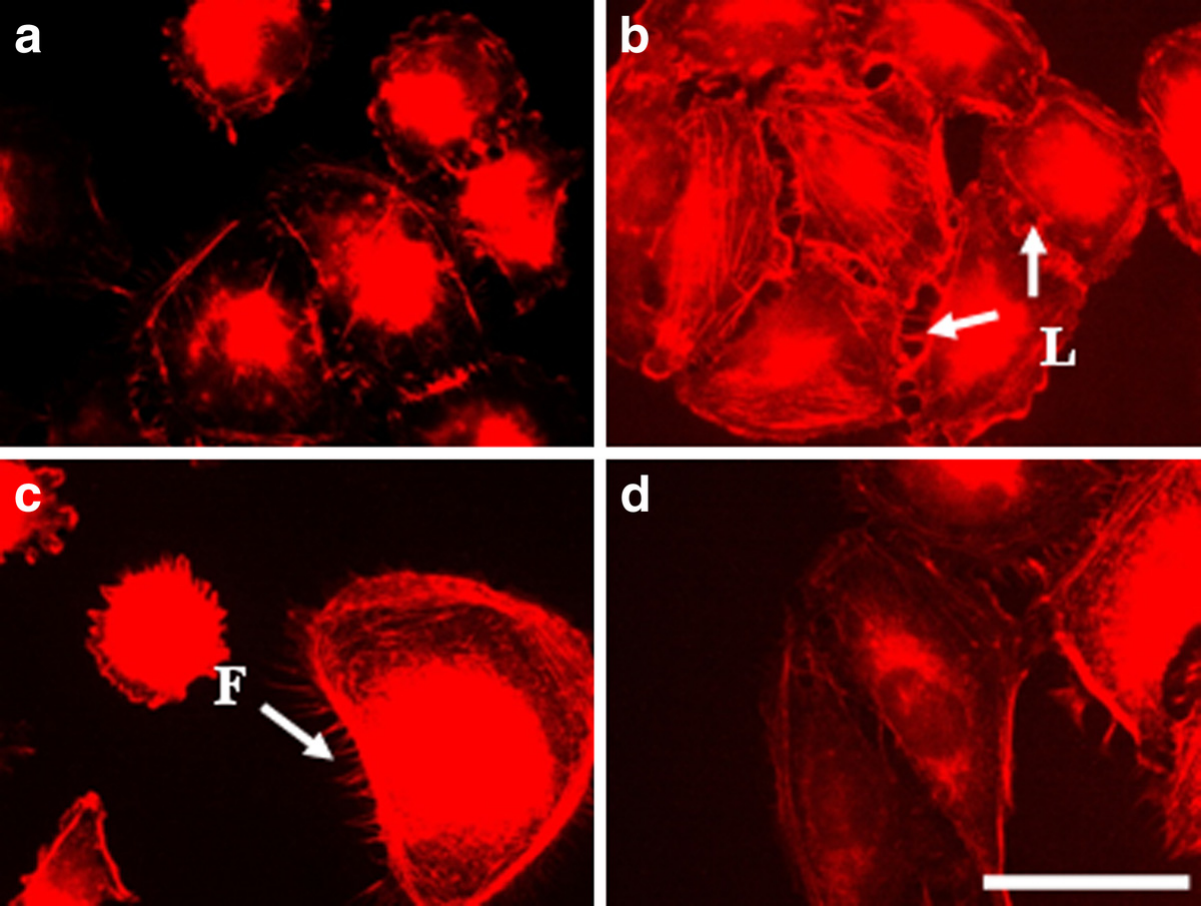
**MCF-7 cells incubated 2 h after electroporation and stained for actin**. (**A**) control, (**B**) electrotreated cells with 200 V/cm, (**C**) with 500 V/cm, (**D**) with 1000 V/cm. L - lamellipodia, F - Filopodia. Bar is 50 μm.

**Figure 13:**
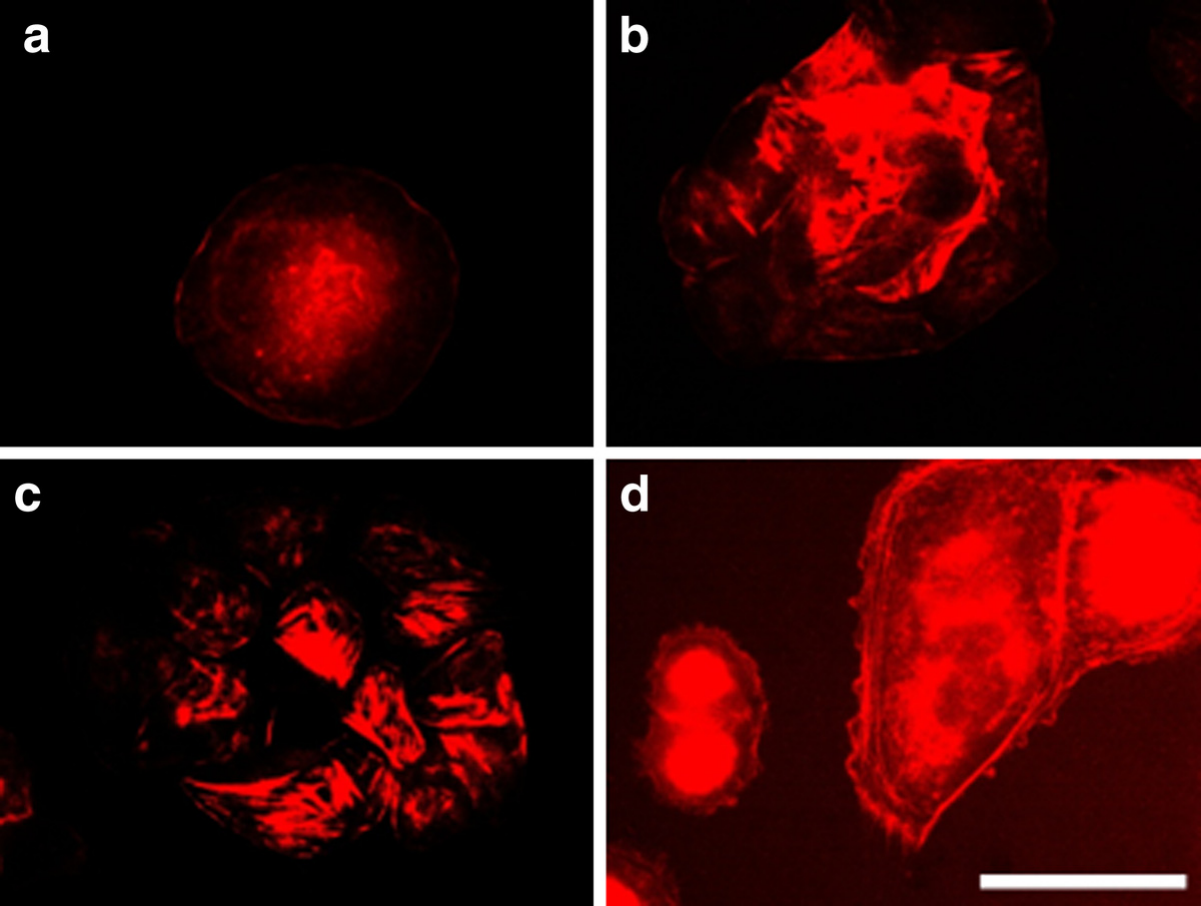
**MCF-7 cells incubated 24 h. after electroporation and stained for actin.** (**A**) control, (**B**) electrotreated cells with 200 V/cm, (**C**) with 500 V/cm, (**D**) with 1000 V/cm. Bar is 50 μm.

**Figure 14:**
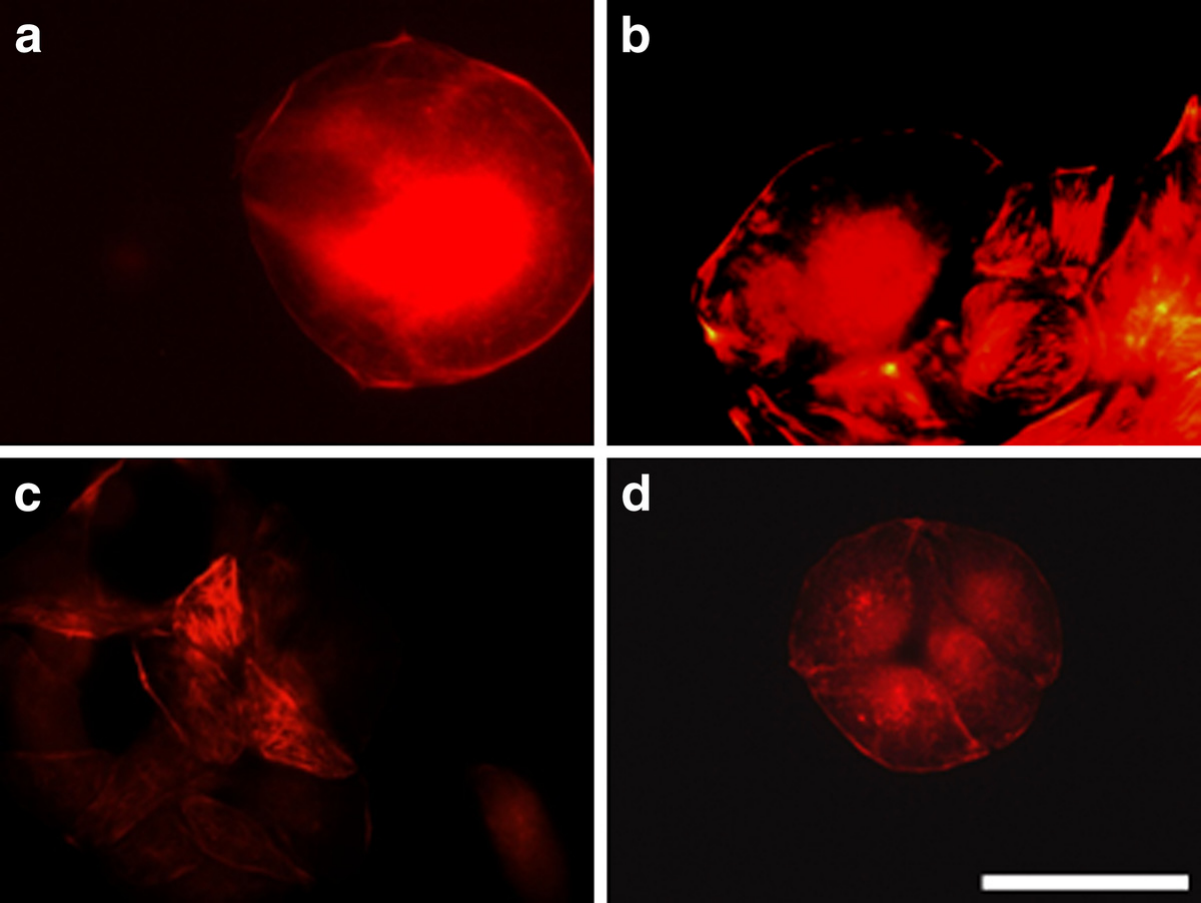
**MCF-7 cells incubated 48 h. after electroporation and stained for actin.** (**A**) control, (**B**) electrotreated cells with 200 V/cm, (**C**) with 500 V/cm, (**D**) with 1000 V/cm. Bar is 50 μm.

By treating of MCF-7 cells with electrical pulses (200 - 1000 V/cm) we also observed changes in the actin cytoskeleton (Figure 12B-D). The cells treated with 200 V/cm revealed well-formed actin stress fibers and cell contacts as well as presence of lamellipodia and filopodia (Figure 12B). The cells treated with 500 V/cm showed also well pronounced filopodia (Figure 12C), while the cells treated with 1000 V/cm (Figure 12D) kept the closest morphology to the control. The tendency for the formation of cell-cell contacts and aggregates appeared 24 hours after electrotreatment for cells treated with 200 V/cm and 500 V/cm (Figure 13B and 13C). After 48 h the process of aggregate formation (cell-cell contacts) was retained and appeared in all the investigated electric field intensities (Figure 14B-D). That fact gives grounds to conclude that for electroporated MCF-7 cells cell-cell contacts are predominant and then cell-substrate contacts followed. Most probably, these cells become more loosely attached to the substratum with time.

In general, for cancer cells electroporation did not cause a significant disturbance in actin cytoskeleton structures. Well visible actin stress fiberes as well as lamellipodia and filopodia could be seen in electroporated cancer cells predominantly on the lower and middle field intensity. That fact could be positive to provoke cell death by application of electrical field as Xiao et al., [26] suggested. In his work he showed that the destruction of the cell cytoskeleton of tumor cells prevents them from necrosis and apoptosis when electrical field is applied.

It is important to note that according to the obtained results for the actin organization after electroporation for MDA-MB-231 cells became predominant after a time cell-substrate adhesion contacts, since electrotreated MCF-7 cells expressed preferentially cell-cell contacts. Thus, it could be assumed that the type of cell adhesion (cell-substrate or cell-cell adhesion) induced by the electroporation process is cell specific. As other investigators [53] have found out that fact could be a result of the involvement of different signal pathways and signal molecules in the process of cell adhesion. Using different signalling inhibitors Wang and colleagues [53] described the ability of invasive and metastatic breast cancer cells either to be reverted to a near-normal phenotype or to cause cell death. Based on the received results we could suggest that the application of high voltage electrical pulses to the transformed MDA-MB-231 cells could lead to their regression to a less or non-transformed cell phenotype since the consolidation of cell-substrate contacts leads to a reduction of cell motility and invasiveness [54].

Also, in MCF-7 cells, an alteration in cell adhesiveness and cell phenotype is observed but the changes are related to the decreased cell-substrate contacts and amplified the ability of the electroporated cells to form stable cell aggregates and cell-cell contacts. It can be suggested that this tendency could lead to a formation of a cell phenotype with decreased cell motility and invasiveness.

## Conclusions

In this article, we provide evidence of the influence of an applied high voltage biphasic external electrical field on the adhesive behaviour of breast tumour cells and fibroblasts. The parameters of the applied electrical field were chosen to be typical for those in electrochemotherapy or lower. The idea was to study the pure effect of the electroporation on cell adhesiveness and actin cytoskeleton. In relation to that, we have several findings:

1. Cell adhesion and survival of fibroblasts and MCF-7 are not affected significantly by the applied electroporation. While, the electrotreatment of invasive breast cancer cell line MDA-MB-231 induces an increase in cell adhesion at lower field intensities and decreased cell adhesion at 1000 V/cm. The cell replication by both cancer cell lines is disturbed by electropermeabilization.

2. Actin cytoskeleton is differently influenced by electroporation in fibroblasts and cancer cells. In 3T3 cells actin cytoskeleton is temporary disturbed, since in cancer cells treated with lower and middle field intensities actin cytoskeleton is well presented in stress fibers, lamelopodia and fillopodia.

3. The type of cell adhesion (cell-substrate or cell-cell adhesion) induced by the electroporation process is cell specific. While by MDA-MB-231 dominates the cellsubstrate adhesion, in MCF-7 cells cell-cell adhesion is more often found.

The present work deals with the changes in adhesive behaviour of fibroblasts and transformed cell lines during the process of electroporation. It raises many questions concerning which signalling pathways are involved in cell adhesion in breast cancer cells and how they can be influenced and/or blocked in order to reach a phenotypic reversion of cancerous cells. The obtained results can be helpful for understanding and multiplying the effect of electrochemotherapy by choosing the suitable electrical parameters.

## Competing interests

The authors declare that they have no competing interests.

## Authors' contributions

VP carried out the electroporation of cells, assay of determination of electroporation, crystal violet assay and immunostaining. IT participated in its design and coordination and helped to draft the manuscript. RT participated in the design and coordination of the study. IT and RT were responsible for interpretation of the data and revision of the manuscript. All authors read and approved the final manuscript
